# A structural-based statistical approach suggests a cooperative activity of PUM1 and miR-410 in human 3'-untranslated regions

**DOI:** 10.1186/1758-907X-1-17

**Published:** 2010-09-22

**Authors:** Limor Leibovich, Yael Mandel-Gutfreund, Zohar Yakhini

**Affiliations:** 1 Department of Computer Sciences, Technion - Israel Institute of Technology, Technion City, Haifa 32000, Israel; 2 Faculty of Biology, Technion - Israel Institute of Technology, Technion City, Haifa 32000, Israel; 3 Agilent Laboratories Israel, Azorim Business Park, 94 Em Hamoshavot Road, 49527 Petach-Tikva, Israel

## Abstract

**Background:**

Micro (mi)RNAs comprise a large family of small non-coding RNAs that are thought to regulate a large fraction of protein-coding genes. Generally, miRNAs downregulate messenger (m)RNA expression by binding to the 3' untranslated regions (UTRs) of the RNA molecules. An important factor for binding specificity is the matching in the seed region. In addition, target site accessibility is thought to be crucial for efficient repression of miRNA targets. Several recent studies indicated that miRNA repression can be facilitated by RNA-binding proteins. In this study, we examine the conjecture that RNA-binding proteins are involved in ushering miRNAs to bind targets that are initially less accessible.

**Results:**

We analyzed human 3'-UTR sequences containing potential binding sites of 153 conserved miRNA families, and ranked sequences around the sites according to their miRNA accessibility. By applying a rank-based motif search tool to these miRNA targets, we found motifs that are enriched among less accessible targets. As expected from our ranking method, most of the significant motifs were GC-rich. However, one AU-rich motif was found to be enriched among miR-410 less accessible targets. This motif resembles the Pumilio homolog 1 (PUM1) consensus binding site. We observed a stronger enrichment of the PUM1 motif in conserved targets than in non-conserved targets; moreover, the enrichment of this motif was found to be conserved in a subset of placental mammals. Further, we analyzed publicly available gene expression data, and found that the mutual expression of PUM1 and miR-410 has a greater negative influence on the expression of low accessibility targets than on other targets, an effect that was stronger than when considering both miR-410 and PUM1 separately.

**Conclusions:**

Taken together, our findings suggest a cooperative relationship between miR-410 and PUM1 in regulating human highly structured 3'-UTRs. This kind of cooperation can allow a second level of regulation of such targets. Considering cases in which miRNAs bind low accessibility targets may help to improve current miRNA prediction tools and to obtain a better understanding of the mechanisms underlying miRNA regulation activity.

## Background

Micro (mi)RNAs are small RNA molecules (approximately 22 nucleotides) participating in a large variety of cellular processes in animals, plants and viruses [1-3]. miRNAs act by binding to the 3'-untranslated region (3'-UTR) of messenger (m)RNAs, forming hybrids that consist of the binding site in the 3'-UTR and of the miRNA seed region (positions 2-8 in the miRNA) [4,5]. miRNAs regulate mRNAs through two main mechanisms: mRNA degradation and inhibition of mRNA translation [6]. It has been shown that the match between the mRNA and the miRNA seed region is important for target recognition [1,7]. However, the number of nucleotide matches in the seed is not the only factor that determines site functionality, and other factors such as site accessibility influence the target recognition [8].

Several recent reports have demonstrated that miRNA repression can be reversed or modulated by RNA-binding proteins (RBPs) interacting with the 3'-UTR of target mRNAs. It was reported that the RNA-binding protein ELAVL1 (embryonic lethal abnormal vision-like protein 1; also known as HuR) reverses miR-122 repression of *SLC7A1 *(also known as *CAT-1*) mRNA in human hepatocarcinoma cells subjected to stress [9]. This effect on *SLC7A1 *is mediated by ELAVL1 translocation from the nucleus to the cytoplasm upon stress, and is accompanied by *SLC7A1 *release from processing (P) bodies, structures involved in RNA metabolism, leading to active translation of the message. The process requires the association of ELAVL1 with AU-rich sequences in the 3'-UTR region of the *SLC7A1 *mRNA, through an as yet unknown mechanism. RBP modulation of miRNA-mediated repression has also been reported for dead end homolog 1 (DND1). In zebrafish, it alleviates miR-430 repression of *nanos1 *and *tdrd7 *in primordial germ cells [10,11]. DND1 can also relieve the repression of cyclin-dependent kinase inhibitor 1B (*CDKN1B*) mediated by miR-221 and the repression of *LATS2 *by miR-372 in HEK293T cells [10]. In zebrafish and in humans, DND1 counteracts miRNA-mediated repression by binding to uridine-rich regions located near the miRNA binding sites within the 3'-UTR of the message. DND1 binding to these sequences might interfere with miRNA-mRNA interaction. Another indication of the functional relationship between miRNAs and RBPs was found in the rat hippocampal neurons, for which treatment with brain-derived neurotrophic factor (BDNF) was shown to partially relieve miR-134 mediated repression of *Limk1 *[12]. When miRNAs regulate mRNAs, they are assembled into ribonucleoprotein complexes known as the miRNA-induced silencing complex (miRISC) [2]. In contrast to ELAVL1, DND1 and BDNF, which relieve the miRNA repressive function, the TRIM-NHL protein family (NHL-2 in *Caenorhabtidis elegans *[13] and TRIM32 in mouse [14]) increase the activity of specific miRNAs, including let-7, by binding to miRISC components.

Pumilio family (PUF) proteins constitute a highly conserved family of RNA-binding proteins that regulate target mRNAs via binding to their 3'-UTRs [15]. PUF proteins are vital in developmental processes, including stem cell maintenance [16-18]. They are also required for long-term memory, and control neuron excitability and development [19-21]. PUF proteins bind specific RNA sequences in 3'-UTRs that contain a core 'UGUR' tetranucleotide followed by sequences that vary between members of this family. mRNA-PUF protein complexes are thought to trigger translational repression or promote mRNA degradation [22-24]. PUF proteins have been recently shown to be associated with miRNAs. It was observed that predicted miRNA binding sites are enriched among validated PUF targets near PUF-binding motifs in humans [25]. In *C. elegans*, the Pumilio homolog PUF-9 is suggested to cooperate with let-7 family members to repress *hbl-1 *in the hypodermis and the ventral nerve cord [26]. This repression requires a region of the *hbl-1 *3'-UTR that contains binding sites for PUF and let-7.

Overall, many examples suggest extensive crosstalk between RBPs and miRNAs. It is likely that additional cases of RBPs modulating miRNA interactions exist. Because efficient repression of miRNA targets is strongly dependent on site accessibility [8], RNA-binding proteins might function as ushers that mediate the opening of the structure, thereby allowing interaction between miRNA and its low-accessibility targets. In this study, we describe a computational approach to seeking evidence for such a mechanism. The approach makes use of a statistical process that includes thermodynamics-based ranking. We highlight one of the cases for which we found significant evidence. Our findings suggest a cooperative mechanism associating the RNA-binding protein Pumilio homolog 1 (PUM1) with miR-410 targeting of low-accessibility target sites in human 3'-UTRs. We found enrichment of the PUM1 binding motif in less accessible miR-410 targets. This association between miR-410 and PUM1 in the context of low-accessibility targets was also significant in other placental mammals (chimpanzee and horse). Furthermore, as a sequence-independent test, we analyzed publicly available gene expression data. We found an inverse relationship between the mutual expression profile of PUM1 and miR-410, and between the expression profiles of the least accessible targets. This inverse relationship was significantly stronger for the combination of PUM1 and miR-410 than for each of them separately. To summarize, by demonstrating a significant association between PUM1 binding sites and highly structured miR-410 targets, our findings suggest that this pair of RBP and miRNA may work together to repress low accessibility targets. Further experiments will be needed to prove this suggested mechanism.

## Results

Our conjecture in this work was that RNA-binding proteins might assist miRNAs in their repression of low-accessibility miRNA targets, with the RBP binding mediating the opening of the secondary structure, thus allowing the miRNA to access the mRNA. This kind of cooperation between RBP and miRNA requires a region of the target 3'-UTR to contain binding sites for both RBP and miRNA. The approach we developed for the exploration of such cooperative pairs of RBP and miRNA is described below.

### Approach description

**Figure 1:**
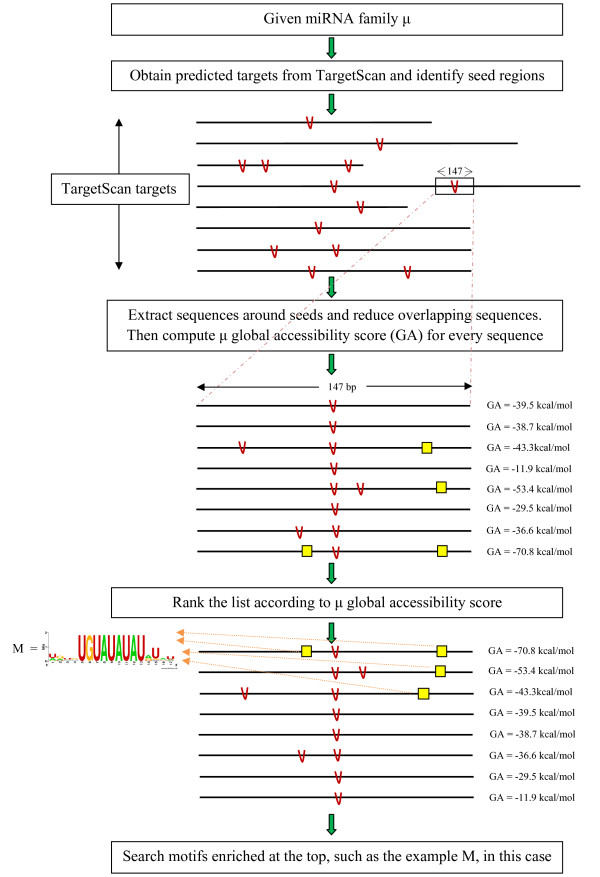
**Identification of motifs enriched among low-accessibility targets of micro (mi)RNA family μ: the complete process**. Given a miRNA family and its binding sites in 3'-untranslated regions (predicted by TargetScan), we extracted sequence elements such that the seed binding site (red V in the figure) was placed in the middle surrounded by 70 base pairs upstream and downstream. We ranked these sequences according to the global accessibility score of μ, and searched for enriched motifs. The motif UGUAUAUAU was found to be enriched among miR-410 low-accessibility targets.

To seek cooperative mechanisms for miRNA activity from sequence data, we implemented a computational process as follows (described schematically in Figure 1):

1) For a given miRNA family μ, we obtained all the conserved predicted targets of μ, including positions of the seed in the 3'-UTR. The predictions were taken from TargetScan [27-29].

2) For every seed occurrence (red V in Figure 1), we considered 70 base pairs on each side.

At the end of this step, we obtained a collection of sequences of length 147 bp with the binding site for μ in the middle, denoted as S_1_,..,S_L(μ)_.

3) To reduce overlap of sequences, we reduced the collection containing S_1_,..,S_L(μ) _using a maximal independent set algorithm (see Methods).

We performed this process for every conserved miRNA family, defined according to [29]. Consequently, for every conserved miRNA family we obtained a set of minimally overlapping conserved sequences containing the predicted target site at the centre of the sequence. The sequences contained the miRNA seed binding site surrounded by 140 bases (70 upstream and 70 downstream), which is sufficient for a reliable prediction of local secondary structures [8].

To detect sequence elements playing a role in structure-driven cooperation with the miRNA of interest given a target sequence S_i_, we defined a criterion that reflects miRNA accessibility to S_i_. Accessibility is reflected by the global accessibility score of each miRNA family to each S_i_. The score takes into consideration both the accessibility of the entire target sequence and the local accessibility of the miRNA binding site (for more details, see Methods). We ranked the target sequences of the miRNA family according to miRNA accessibility, with the least accessible targets located at the top of the list and the more accessible targets ranked lower in the list. Furthermore, to avoid motifs that overlap with the miRNA binding site or with its reverse complement, we masked the miRNA binding site (located at the centre of the sequence) and the nucleotides that are predicted to form base pairs with it. We next sought motifs enriched among the least accessible targets in comparison with the accessible targets. This was done using DRIM [30], which was adapted for finding RNA motifs. To reduce the number of false positive results, we concentrated on relatively long motifs of length 9 bp.

### The Pumilio binding motif is enriched in miR-410 low accessibility targets

To search for RNA-binding motifs that may be associated with low accessibility target sites, we repeated the process described above for the 153 conserved miRNA families known for humans [29]. We found 163 enriched motifs for the 153 miRNA families that passed the minimum hypergeometric (mHG) score threshold of 10^-4 ^(see Methods). Every motif is associated with a miRNA family μ, such that it is enriched among the least accessible targets of μ. As expected from our accessibility score and ranking approach, sequences appearing at the top of the ranked list tended to have greater GC content than did those located at the bottom of the list (see Additional file 1, Figure S1). Therefore, we expected to find enrichment of GC-rich motifs in low-accessibility targets.

**Figure 2:**
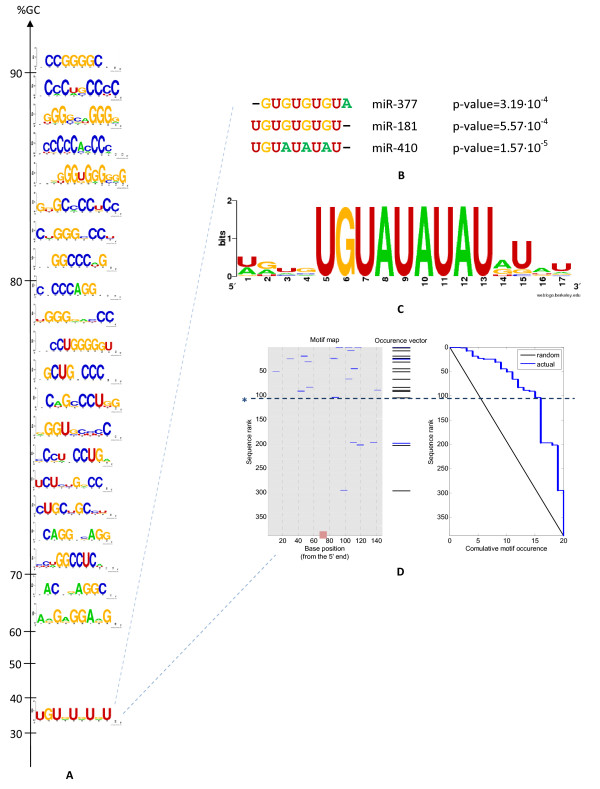
**Clustered motifs found to be enriched among micro (mi)RNA low-accessibility targets in humans**. **(A) **The motifs found to be enriched among miRNAs low-accessibility targets were clustered according to sequence similarity. Clusters containing at least three motifs are shown. For each cluster, we calculated the average GC content over all motifs in the cluster. The clusters were sorted according to their GC content. Note that the GC content axis is variable. **(B) **The multiple sequence alignment of the motifs belonging to the cluster with the lowest GC content is shown. For each member of the cluster, the miRNA family for which it was found and the enrichment *P *value are mentioned. **(C) **The logo of the motif enriched among miR-410 least accessible targets is shown. This motif holds the lowest GC content found. **(D) **On the left, the occurrences of UGUAUAUAU among miR-410 predicted targets are shown. The targets were ranked according to the miR-410 global accessibility score. The location of the miRNA binding site is marked with a pink rectangle on the *x *axis. In the middle, the occurrences vector plot illustrates the number of motif occurrences in each sequence (black for one occurrence, blue for two occurrences). On the right, the actual motif accumulated occurrences versus the expected motif accumulated occurrences in a random dataset are shown, highlighting the observed enrichment. The dashed line (and asterisk) indicates the minimum hypergeometric (mHG) cutoff used for partitioning the sequences into two subsets (such that the motif is enriched in the upper subset).

Furthermore, we clustered the identified motifs into groups based on sequence similarity, considering only clusters containing ≥ 3 motifs (Figure 2A). As control, we conducted the process described above for *Saccharomyces cerevisiae *3'-UTRs, working with human miRNAs and using a similar number of targets as in humans. Because the miRNA machinery is not known to exist in *S. cerevisiae*, we considered the results we obtained here as being random (or as negative control). In the *S. cerevisiae *control, we found 33 enriched motifs versus 163 motifs in humans (at mHG ≤ 10^-4^). Clustering the *S. cerevisiae *motifs yielded clusters containing only one or two motifs (see Additional file 1, Figure S2 for the mHG scores of human motifs versus *S. cerevisiae *motifs; for more details on this control, see Methods).

Among the results for humans, we detected one exceptional cluster having a relatively low GC content (the multiple sequence alignment for this cluster is shown in Figure 2B). In this cluster, the motif found for miR-410 (UGUAUAUAU) contained only one G (11.1% GC). Interestingly, no motif having such low GC content was found in the *S. cerevisiae *control (at mHG score ≤ 10^-4^). This motif perfectly contains the consensus binding site of PUM1 and PUM2, which is UGUAHAUA [25,31], suggesting an association between the RNA-binding proteins of the Pumilio family and the miR-410 low accessibility targets (the motif is shown in Figure 2C, its enrichment among miR-410 low accessibility targets is illustrated in Figure 2D, and the low accessibility targets containing the motif are listed in Table 1; see Additional file 1, Figure S3 for their structures).

**Table 1:** MiR-410 low accessibility targets containing the motif UGUAUAUAU.

Gene symbol	Gene name	PANTHER molecular function
*FAM120C*	Family with sequence similarity 120C	Unclassified
*C14orf102*	Chromosome 14 open reading frame 102	Unclassified
*NRIP1*	B-cell CLL/lymphoma 11B (zinc finger protein)	Zinc finger transcription factor Nucleic acid binding
*DGKG*	Diacylglycerol kinase, gamma 90 kDa	Kinase
*RAI1*	Retinoic acid induced 1	Transcription factor
*MCAM*	Melanoma cell adhesion molecule	CAM family adhesion molecule
*STARD13*	START domain containing 13	Other G-protein modulator
*CELSR1*	Cadherin, EGF LAG seven-pass G-type receptor 1 (flamingo homolog, Drosophila)	G-protein coupled receptor Cadherin
*ZC3H7B*	Zinc finger CCCH-type containing 7B	Nucleic acid binding
*NDST1*	*N*-deacetylase/*N*-sulfotransferase (heparan glucosaminyl) 1	Other transferase
*FZD5*	Frizzled homolog 5 (*Drosophila*)	Molecular function unclassified
*HACE1*	HECT domain and ankyrin repeat containing, E3 ubiquitin protein ligase 1	Ubiquitin-protein ligase
*TEC*	Tec protein tyrosine kinase	Non-receptor tyrosine protein kinase
*PRRX1*	Paired related homeobox 1	Homeobox transcription factor Other DNA-binding protein

Fourteen genes at the subset of top 105 low accessibility targets contain the motif UGUAUAUAU. Their gene symbols, gene names and PANTHER [49] molecular functions are shown.

### Controls

To further study the proposed association, we conducted a list of control experiments, described below. Previous studies have shown that miRNA binding sites are occasionally found in multiple copies in 3'-UTRs [32,33]. Multiplicity of miRNA binding sites has been suggested to be correlated with degree of repression. Because the presence of multiple binding sites of miR-410 in the 3'-UTRs could influence our results, we checked whether binding site multiplicity was correlated with our ranking for miR-410 targets. We found that miR-410 binding site multiplicity did not correlate with the accessibility ranking of miR-410 predicted targets (Pearson correlation coefficient = 0.062), thereby ruling out multiplicity as a driver for the aforementioned enrichment (for more details, see Methods).

An alternative explanation for the observed enrichment of the Pumilio motif among low accessibility miR-410 targets is that Pumilio binding sites are generally more prevalent in highly folded targets. To test this, we calculated the enrichment of the Pumilio consensus motif [25,31] in UTR sequences ranked according to ΔG calculated from the predicted secondary structure. Interestingly, we did not observe any enrichment of the Pumilio motif in highly structured UTRs (*P *= 0.24 for the best enrichment among 100 repetitions; for more details, see Methods). These results reinforce the association between miRNA accessibility and the observed enrichment of the Pumilio motif in the least accessible miR-410 targets.

To examine whether Pumilio binding sites are generally enriched among GC-rich sequences, we ranked the predicted targets of each miRNA family in our list of conserved miRNA families according to their GC content, and recalculated the enrichment of the Pumilio consensus [25,31]. Among the 153 conserved miRNA families (including miR-410), the best enrichment found for the Pumilio motif had a *P *value of 0.21, thus excluding this explanation.

Furthermore, as a control for our ranking approach, we took the 100 least accessible targets and 100 most accessible targets (of miR-410) and searched for enriched motifs in each subset, using MEME [34]. We masked the miRNA binding site and its complement to avoid enrichment of motifs derived from the miRNA binding site. In the subset of least accessible targets, the most enriched motif found was an AU-rich motif that is similar to the Pumilio motif (its regular expression is AU[AG][UC]AUAUAUAUAUA; e-value = 1.4 × 10^-30^). This motif (or any similar motif) was not found for the subset of accessible targets. Moreover, the best e-value per motif in the latter subset was 7.3 × 10^-6^.

To assess whether the observed association between the Pumilio motif and the predicted, least accessible miR-410 sites could reflect a functional relationship between the RBP and the miRNA, we generated a subset of predicted miR-410 targets that are evolutionarily conserved and thus more likely to be functional miR-410 sites [35]. We then compared the enrichment of the Pumilio motif among predicted targets of human miR-410 taken from three datasets: conserved targets, targets with no restriction on conservation, and non-conserved targets. We found that Pumilio was most significantly enriched in the conserved dataset (*P* = 1.57 × 10^-5^), whereas it was less enriched in both the miR-410 predicted targets with no restriction on conservation (*P* = 2.15 × 10^-3^) and in the non-conserved dataset (*P* = 4.2 × 10^-2^; for more details, see Methods). The higher enrichment of Pumilio binding sites among the conserved, least accessible miR-410 targets may indicate that this association is related to miRNA function.

To further validate that the Pumilio motif is functionally related to miR-410, we used TargetScan to predict a set of miR-410 pseudo-targets (computed based on sequence match with the miRNA seed) in the 3'-UTRs of organisms lacking miR-410 (*C. elegans*) or miRNAs in general (*S. cerevisiae*). We then calculated the enrichment of the motif among low accessibility targets for each organism (using broadly the same process described above). As expected, the Pumilio motif was not found to be enriched in any of these organisms (*S. cerevisiae*: *P *= 0.35, *C. elegans*: *P *= 0.76). Next, we applied the latter test to placental mammals in which miR-410 is conserved [29]. For this analysis, we used the dataset of conserved targets predicted by TargetScan for chimpanzee, horse and dog. Here, again, the Pumilio motif was found to be enriched among miR-410 low-accessibility targets, specifically in chimpanzee (*P *= 5.28 × 10^-4^) and horse (*P *= 1.4 × 10^-4^), but it was only weakly enriched in dog (*P *= 3.3 × 10^-2^). It is important to note that these organisms were chosen because the sizes of their datasets are very similar to that of the human dataset, and thus the differences in *P *values cannot be due to the size of the datasets.

### Additional controls using validated Pumilio targets in humans

To further investigate the relationship observed between miR-410 and the Pumilio family and to show its dependence on target accessibility, we used experimental Pumilio binding data available for humans [25]. We obtained 3'-UTR sequences of validated Pumilio targets and ranked them according to their corresponding affinity to Pumilio, as reported previously [25]. We then calculated the enrichment of the miR-410 binding site among validated Pumilio targets. The miR-410 binding site was not found to be enriched among Pumilio targets (PUM1: *P *= 0.86, PUM2: *P *= 0.48). As a control, we calculated the enrichment of the Pumilio recognition motif (UGUAHAUA [25,31]) among validated Pumilio targets, and found it to be strongly enriched among PUM1 targets (*P *= 2.47 × 10^-9^) and weakly enriched among PUM2 targets (*P *= 2.7 × 10^-2^). To summarize, the analysis presented here demonstrates that the miR-410 binding motif is not generally enriched in Pumilio targets (see Additional file 1, Figure S4). These findings indicate that the observed association of Pumilio and miR-410 cannot be explained by a general association between them. They therefore support a more specific role of Pumilio, closely associated to low-accessibility miR-410 targets.

Next, we evaluated the association of Pumilio proteins with other miRNAs in the context of low-accessibility targets using the knowledge of experimentally validated Pumilio targets [25]. We used mHG statistics to calculate the enrichment of validated Pumilio targets in the list of miRNA targets ranked according to global accessibility (see Methods). We did this for all 153 human miRNA families. Of the 153 experiments, the most significant enrichment was found for miR-410 (*P *= 1.4 × 10^-2^; 14 out of 388 miR-410 targets are validated Pumilio targets and all are in the list of the top 251 least accessible targets).

### PUM1 cooperation with miR-410 based on gene expression analysis

The aforementioned experiments suggest the involvement of the Pumilio family in modulating miR-410 repression of low-accessibility targets. In this section, we used gene expression data (on the NCI60 panel [36,37]) to test PUM1 cooperation with miR-410.

**Figure 3:**
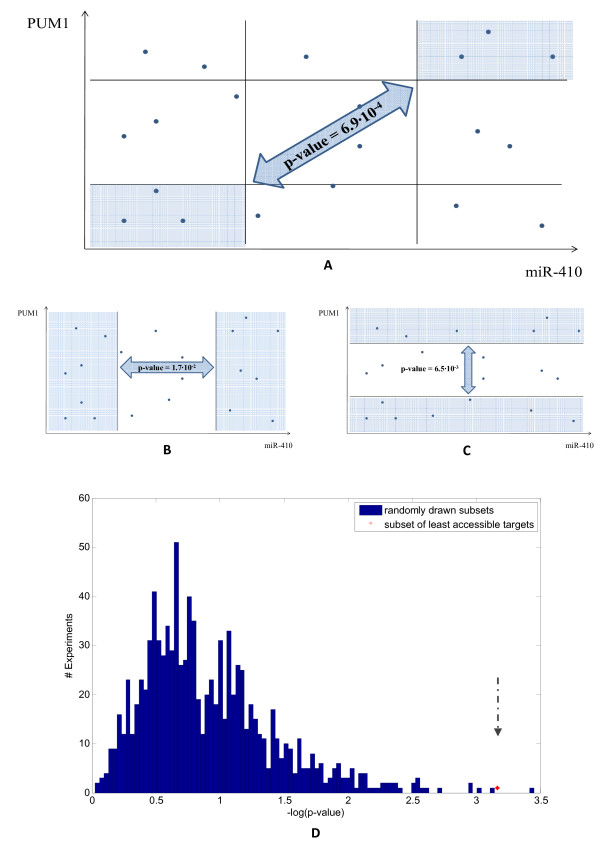
**Cooperative repressive influence of PUM1 and miR-410 on least accessible targets**. **(A) **In this test, we compared samples having high levels of PUM1 and miR-410 expression with samples having low expression, and calculated the degree of differential repression for all targets. We found that the set of least accessible targets was significantly differentially repressed compared with other targets (*P *= 6.9 × 10^-4^). Note that the expression values of PUM1 and miR-410 are drawn schematically. **(B,C) **We repeated the test described in (A) but now ignored PUM1 and miR-410, respectively. In each of these tests, the differential repression of the least accessible targets compared with other targets was weaker than in (A) (*P *values obtained are indicated in the figure). **(D) **We repeated the test described in (A) for 1,000 randomly drawn subsets instead of the subset of least accessible targets. Each random subset contained the same number of targets as the subset of least accessible targets and was disjoint from it. The differential repression of 999 subsets was less significant than 6.9 × 10^-4^.

According to the suggested mechanism, two main observations should hold. First, in the presence of high levels of PUM1 and miR-410, there should be a stronger repression effect on the set of least accessible targets than in the presence of low levels of PUM1 and miR-410. The set of least accessible targets (see Methods) is hereby denoted by Φ. The difference in the extent of repression comparing the two states (defined according to PUM1 and/or miR-410 expression levels) is hereafter termed as 'differential repression'. Differential repression is expected to be more dramatic for Φ than it is for other (more accessible) miR-410 targets. Indeed, the significance of differential repression of the least accessible targets compared with the rest of the targets was 6.9 × 10^-4 ^(for more details, see Methods). The second expected observation is that the differential repression of the least accessible targets, Φ, versus the rest of the targets should be less significant than when partitioning samples according to only miR-410 or only PUM1, owing to the suggested cooperative interaction. Indeed, the mutual differential repression was ~25-fold more significant than when considering only miR-410, and ~10-fold more significant than when considering only PUM1 (Figure 3, A-C).

To validate that the mutual repressive influence of PUM1 and miR-410 is related to the low accessibility of the miRNA targets, we repeated this process 1,000 times, each time for a randomly drawn subset of genes, Φ', taken from the pool of miR-410 predicted targets (each Φ' contains the same number of elements as Φ; each Φ' is disjoint from Φ). As shown in Figure 3D, of the 1,000 experiments, only one result was better than 6.9 × 10^-4 ^(*P *= 3.55 × 10^-4^).

## Discussion

The extent to which miRNAs interact with low accessibility targets is not clear, but if such binding takes place there could be a molecular mechanism allowing the miRNA to bind highly structured targets, possibly by involving RNA-binding proteins. To date, there is increasing evidence for cooperation between miRNAs and RBPs [9,10,12-14,26]; however, the possible role of RBPs in facilitating miRNA binding to inaccessible sites has yet not been examined carefully. In this study, we sought sequence motifs that are enriched among miRNA low-accessibility targets, because these motifs may point to RNA-binding proteins that take part in this kind of mechanism. We clustered the motifs found into groups based on sequence similarity (see Methods). The cluster containing the motif UGUAUAUAU that was found for miR-410 (Figure 2B) was exceptional because of its low average GC content (33%). The above motif was especially interesting because it is the well-characterized Pumilio recognition motif [25,31] and was unusually AU-rich. The other motifs in the cluster comprised UG runs known as binding motifs of the heterogenous RNP protein trans-activation-response DNA binding protein (TARDBP; also known as TDP-43) [38].

Testing the enrichment of the Pumilio motif among miR-410 low-accessibility targets using various controls strongly supported the conjecture that this association is related to the miRNA accessibility of the targets. In addition, the accessibility-dependent association between PUM1 and miR-410 was found to be conserved in humans, chimpanzee and horse, and it was more significant in the human conserved dataset than in the human non-conserved dataset, implying functionality. Moreover, analyzing publicly available gene expression data revealed that mutual expression of PUM1 and miR-410 has a greater negative influence on low-accessibility targets than on other targets. Interestingly, mutual expression of PUM1 and miR-410 had a greater negative influence than did their individual influences separately, supporting our conjecture that miR-410 and PUM1 act together, possibly by facilitating the repression of low-accessibility miR-410 targets.

In a previous study by Fiore *et al*. [21], it was demonstrated that the miRNA cluster miR379-410 (containing miR-410) is transcriptionally activated upon activation of mouse cortical neurons, and that one component of this cluster, miR-134, takes part in triggering activation-dependent dendritogenesis. The latter study further suggests that *Pum2 *is a miR-134 target in this process. Based on the verified cooperation between Pumilio and the miR379-410 cluster in dendritogenesis, we speculate that the cooperation between miR-410 and Pumilio suggested in this study might be part of the process regulating dendritogenesis. Clearly, further experimental assays should be carried out to validate this observed association of miR-410 and PUM1 in human 3'-UTRs, and to explain the related mechanisms.

## Conclusions

An association between miRNAs and Pumilio was suggested previously [25], based on the observation that predicted miRNA binding sites are enriched among validated Pumilio targets near Pumilio binding motifs. In this work, we did in fact observe an association, specific to the context of low accessibility, between Pumilio binding sites and miR-410 targets. We suggest that PUM1 and miR-410 may cooperate in repressing highly structured targets, allowing a second level of regulation of these targets. We hypothesized a mechanism in which PUM1 plays a role in ushering miR-410 to highly structured targets. It is likely that additional pairs of miRNAs and RBPs, as yet undiscovered, cooperate in a similar way. Taking into account the possibility that miRNAs can bind low-accessibility targets with the assistance of RNA-binding proteins may help in improving the accuracy of miRNA target prediction tools and in identifying novel regulatory mechanisms.

## Methods

### NCI60 dataset

This dataset comprised a panel of 60 cancer-derived cell lines. For each of the 60 cell lines, we obtained mRNAs and miRNA expression data from the literature [36,37].

### Human validated Pumilio targets dataset

Lists of PUM1 and PUM2 target mRNAs in HeLa S3 cells were obtained [25]. The dataset included gene information and numerical data related to the measured affinity between Pumilio proteins and the potential targets.

### Maximal independent set

Given a set of sequence elements S_1_,..,S_L_, which are substrings of a gene 3'-UTR, there can be overlap between sequences. Each S_i _is associated with two coordinates that define its start and its end positions in the 3'-UTR. To reduce overlap between sequences, given S_i _and S_j_, we required that start(S_i_)+100 be less than or equal to start(S_j_) (that is, that the distance between the starts of every two sequences in the set must be greater than 100 nucleotides). Consider the interval graph [39] G = (V,E), whose set of vertices is V = {S_1_,..,S_L_} and set of edges is E = {(S_i_,S_j_) | i < j and start(S_i_)+100 > start(S_j_)}. Without loss of generality, the sequences are sorted according to their starting positions, such that the starting point of S_1 _is minimal. To find a maximal set of minimally overlapping sequences, we need to find a maximal independent set in G. Because G is an interval graph, the optimal solution can be found efficiently [39].

### TargetScan

TargetScan predicts the biological targets of miRNAs by searching for the presence of conserved eight-mer and seven-mer sites that match the seed region of each miRNA [27-29]. As an option, non-conserved sites are also predicted. Sites with mismatches in the seed region that are compensated for by conserved 3'- pairing are also identified [29]. We used TargetScanHuman [40], which considers matches to annotated human UTRs and their orthologs.

TargetScan provides a code that enables making custom predictions of miRNA binding sites for any arbitrary given set of syntactically valid sequences. We used it to obtain predictions in a variety of organisms (such as *S. cerevisiae*, even if they lack miRNA activity).

Conserved miRNA families were defined according to previously published data [29]. They included broadly conserved families (which are conserved across most vertebrates, usually as far as zebrafish) and conserved families (which are conserved across most mammals, but usually not beyond placental mammals).

### RNAfold and miRNA global accessibility score

RNAfold is a software application that predicts the secondary structures of single-stranded RNA or DNA sequences [41].

Given a miRNA family μ and a target sequence S_i_, we defined the miRNA global accessibility criterion for S_i _as follows:

• We calculated the free energy of the entire sequence, denoted as ΔG_all_(S_i_).

• We calculated the free energy of S_i _when the area surrounding the seed binding site is forced to be unpaired, denoted by ΔG_masked_(S_i_).

• The free energy lost in opening the structure at the binding site of μ in S_i _was then defined as:

$$\Delta {\text{G}}_{\text{open}}\left({\text{S}}_{\text{i}}\right)=\Delta {\text{G}}_{\text{all}}\left({\text{S}}_{\text{i}}\right)-\Delta {\text{G}}_{\text{masked}}\left({\text{S}}_{\text{i}}\right).$$

This number reflects the local accessibility of the miRNA binding site; the more negative this value, the greater the energy required for opening the target site secondary structure.

• The global accessibility of S_i _to μ binding is represented by:

$$\text{GA}\left({\text{S}}_{\text{i}}\right)=\Delta {\text{G}}_{\text{open}}\left({\text{S}}_{\text{i}}\right)+\Delta {\text{G}}_{\text{all}}\left({\text{S}}_{\text{i}}\right)=\text{ 2}\Delta {\text{G}}_{\text{all}}\left({\text{S}}_{\text{i}}\right)-\Delta {\text{G}}_{\text{masked}}\left({\text{S}}_{\text{i}}\right)$$

It should be noted that to calculate ΔG_masked_(S_i_), given a sequence S_i_, we masked the area surrounding the target site in S_i _(25 bases in total, with the miRNA binding site located in the middle) and calculated the free energy of the modified sequence using RNAfold. Technically, putting the letter N in any region in a sequence leads RNAfold to avoid base pairing in that region (see Additional file 1, Figure S5) and thereby masks the region.

### DRIM

DRIM (discovery of rank imbalanced motifs) is a software application that identifies sequence motifs in lists of ranked DNA sequences [30], and it has also been adapted for RNA sequences [42]. DRIM employs a flexible threshold statistical approach [30,43] to discover motifs that are significantly enriched at the top of a ranked list of sequences compared with the rest of the list.

The motifs returned by DRIM are seed motifs that are sufficiently significant to be passed as input to the heuristic search mechanism of DRIM. The default threshold, which is also used here, is mHG score = 10^-4 ^(motifs with greater scores are discarded).

### *S. cerevisiae *control

We downloaded 3'-UTR sequences of *S. cerevisiae *from the UCSC Table Browser [44] and predicted the (imaginary) binding sites of human miRNA families within these UTRs using TargetScan script. To allow the same statistical power as in the human dataset, we used a similar number of targets per miRNA family for the tests described in this paper (if there turned out to be more sequences than needed, we would then filter the sequences in the middle of the ranked list). The number of *S. cerevisiae *targets per miRNA is the same as in humans, except for sporadic cases in which there are fewer targets in *S. cerevisiae *(on average, the difference between the number of targets in humans and the number of targets in *S. cerevisiae *per miRNA is 12).

### Procedure for clustering motifs

Given two sequences s = s_1_,..,s_n _and t = t_1_,..,t_n_, the i-level distance between s and t is defined as i plus the number of mismatches between the sequences s_1_,..,s_n-i _and t_i+1_,..,t_n_.

Given two sequences s = s_1_,..,s_n _and t = t_1_,..,t_n_, the distance between s and t is defined as the minimal i-level distance for i = 0,..,n-1.

Given two sets of sequences C_1 _and C_2_, we defined the distance between C_1 _and C_2 _as the average of distances between every two sequences s and t, such that s ∈C_1 _and t ∈C_2_.

The clustering procedure we applied here is given as its input a set of sequences to cluster and a parameter denoted as diameter. It begins with clusters that are singletons; each sequence is a single cluster. It recursively merges the pair of closest clusters, and halts when the distance between the closest clusters is greater than the diameter given as a parameter. This is a variant of the nearest neighbour hierarchical clustering approach.

To produce the motif Shannon logo for a cluster, we calculated the multiple sequence alignment for the members of the cluster using ClustalW2 [45] and drew the motif using WebLogo [46].

### Multiplicity test

To test whether multiplicity is correlated with our ranking, we counted the number of miRNA binding sites within each predicted target sequence by counting the number of matches of the miRNA seed in the sequence. In addition to Watson-Crick base pairing, we allowed wobble base pairs (G-U). We also allowed one mismatch between the miRNA seed and its binding site and counted overlapping binding sites.

### Testing possible association between Pumilio and ΔG

To calculate the enrichment of Pumilio in highly folded targets, we generated 100 sets of UTR sequences and ranked them according to their ΔG. The length of the sequences and the size of each set were the same as for the miR-410 dataset to allow for the same statistical power. The UTR sequences in each set were chosen randomly.

### Conservation control

In this control, we used three datasets of miR-410 predicted targets in humans: conserved targets, non-conserved targets, and targets with no constraint on conservation. The conserved predicted targets dataset and the non-conserved predicted targets dataset were obtained from TargetScan. For the third dataset, we downloaded human 3'-UTR sequences from the UCSC Table Browser [44], and calculated miR-410 targets within these sequences using TargetScan script. In this control, as in the *S. cerevisiae *control, we used the same number of targets for the three datasets. We calculated the enrichment of the Pumilio motif among the target sequences of each set.

### Enrichment analysis (using mHG statistics)

An approach has been previously described [30] to identify the enrichment of a set of genes, A, in a ranked list of genes using mHG statistics. Given a total number of genes N, with B of these genes belonging to A, and n of these genes being in the target set (for example, differentially expressed genes), the probability that b or more genes from the target set are also in A is given by the hypergeometric tail (HGT):

$$P(X\geq b)=HGT(b;N,B,n)=\sum_{i=b}^{\min(n,B)}\frac{\left(\begin{array}{l} n\\ i \end{array}\right)\left(\begin{array}{l} N-n\\ B-i \end{array}\right)}{\left(\begin{array}{l} N\\ B \end{array}\right)}$$

If a ranked genes list g_1_,..,g_N _is provided in place of a target set, we define a label vector *λ = λ*_1_,...,*λ*_N _∈{0,1}^*N *^according to the association of the ranked genes to A, that is, λ_i _= 1 if and only if g_i _is in A. The mHG score is then defined as:

$$mHG(\lambda )=\underset{1\leq n<N}{\min}HGT(b_{n}(\lambda );N,B,n)$$

where

$$b_{n}(\lambda )=\sum_{i=1}^{n}\lambda_{i}$$

In other words, the mHG score is the optimal HGT probability that is found over all possible partitions induced by the ranking. As such, this score must be corrected for multiple testing. A dynamic programming algorithm for computing the exact *P *value of a given mHG score has been described previously [30]. More specifically, given a ranked list of genes, a subset A, and a corresponding mHG score s, the mHG *P *value tells us the exact probability of observing an mHG score s' ≤ s under the null assumption that all occurrence configurations of A in the ranked list are equiprobable.

### Enrichment of Pumilio experimentally validated targets among low accessibility miRNA targets

The set of validated Pumilio targets contains 1,482 genes (comprising both PUM1 and PUM2 targets). Given a miRNA family denoted as μ, using the mHG statistics we calculated the enrichment of Pumilio targets in the list of μ targets ranked according to their global accessibility scores (such that least accessible targets are at the top) as follows.

1) We used the list of conserved predicted targets, S_1_,..,S_N_, ranked according to global accessibility, GA(S_i_) of μ (the calculation of GA(S_i_) is described above).

2) We produced a binary vector λ(μ) as follows:

For each sequence S_i _in the list of predicted targets for μ, λ_i_(μ) = 1 if and only if S_i _was reported as a Pumilio validated target and also contained the Pumilio consensus motif (UGUAHAUA [25,31]). We required S_i _to contain the Pumilio motif in addition to being a validated target because Pumilio target genes have been reported [25] without an indication of where the binding sites reside in the 3'-UTR. In addition, we required that the Pumilio binding site did not overlap with the predicted miRNA binding site or with its complement.

3) We used the mHG statistics on λ(μ) to calculate the enrichment of validated Pumilio targets in low-accessibility targets of μ.

### Differential expression

To compute the differential expression, we used the threshold number of misclassifications (TNoM) score and *P *value previously described [47].

### Samples classification algorithm (used in the gene expression analysis)

The expression analysis requires having two disjoint subsets (denoted as A and B) such that A contains the samples in which PUM1 and miR-410 are highly expressed, whereas B contains the samples in which PUM1 and miR-410 are expressed at low levels. To identify an appropriate partition for this purpose, we performed a class discovery process. Let the vector *x *= *x*_1_,...,*x*_*N *_be the expression profile of miR-410 in samples 1,..,N and let the vector *y *= *y*_*1*_,...,*y*_*N *_be the expression profile of PUM1 in samples 1,..,N. Because (*x*_*i*_,*y*_*i*_) is the expression of miR-410 and PUM1 in the i^th ^sample, respectively, we can define the set P ⊆ **R**^2 ^that contains *N *points representing the expression of miR-410 and PUM1 in the *N *samples.

We sorted P by constructing a two-dimensional k-dimensional (kd)-tree ([48]) that would follow the sorting order. At the root, we split the set P with a vertical line into two subsets of roughly equal size. P_left_, the subset of points to the left or on the splitting line was stored in the left subtree, and P_right_, the subset to the right of the splitting line, was stored in the right subtree. At the left child of the root, we split P_left _into two subsets with a horizontal line: the points below or on it were stored in the left subtree of the left child and the points above it were stored in the right subtree. Similarly, the subset P_right _was split with a horizontal line into two subsets that were stored in the left and right subtrees of the right child. At the grandchildren of the root, we again split with a vertical line. In general, we split with a vertical line at nodes whose depth was even, and with a horizontal line at nodes whose depth was odd. The algorithm terminates when all the subtrees are leaves. Scanning the leaves from left to right produces the sorted list.

The construction of the kd-tree uses O(*N*) storage and takes O(*N*log*N*) time.

Having the sorted list of points p_1_,..,p_N_, such that p_1 _< p_2 _< ... < p_N_, we defined the following configurations:

$$S_{k}=\left\{(A_{k},B_{k}\text{) }|\;A_{k}=\{p_{N-k+1},...,p_{N}\text{\} }and\;B_{k}=\{p_{1},...,p_{k}\right\}\}\;,\;k=1,...,\left\lfloor \frac{N}{2}\right\rfloor .$$

Given a figure of merit, we calculated the figure of merit for all the configurations and took the configuration S_k _= {A_k_,B_k_} that held the optimal value.

We now explain the figure of merit used in our process. Consider a set Φ of mRNAs of interest (for example, miR-410 least accessible targets). We evaluated the differential expression of Φ due to miR-410 and PUM1 expression levels as follows.

1) Consider a configuration A, B.

2) For every miR-410 predicted target τ we calculated the TNoM *P *value, measuring whether its expression in A was lower than its expression in B (termed as the differential repression of τ).

3) We ranked miR-410 targets according to their TNoM *P *values in increasing order.

4) We calculated the enrichment of the genes of Φ at the top of this ranked list.

5) Finally, we selected the best configuration A, B using the enrichment calculated in the previous step as the figure of merit.

### Definition of least accessible targets

Given a list of miRNA targets ranked according to miRNA accessibility, the set of least accessible targets (denoted as Φ) was defined as the top 20% targets of the ranked list.

## Competing interests

The authors declare that they have no competing interests.

## Authors' contributions

LL, YMG and ZY designed the study. YMG proposed the research question. LL and ZY developed the methodology and the algorithmic approaches. LL developed the software and performed the data analysis. All authors contributed to and read and approved the final manuscript.

## Supplementary Material

Additional file 1 **Supplemental Figures S1-S5**. Supplemental Figure **S1 **- Correlation between rank and GC content calculated for the targets of every micro (mi)RNA family. Supplemental Figure **S2 **- Minimum hypergeometric (mHG) scores of human motifs versus mHG scores of *Saccharomyces cerevisiae *motifs. Supplemental Figure **S3 **- Predicted structures of selected mRNA targets. Supplemental Figure **S4 **- The enrichment of the Pumilio binding motif versus the enrichment of the miR-410 binding motif in validated Pumilio targets. Supplemental Figure **S5 **- Illustration of masking a region in a sequence. Click here for file
