# Supplementary material for: A structural-based statistical approach suggests a cooperative activity of PUM1 and miR-410 in human 3'-untranslated regions
Source: Silence. 2010 Sep 22;1:17. doi: 10.1186/1758-907X-1-17 (PMC2955683; doi:10.1186/1758-907X-1-17)
Supplement: Additional file 1 — Supplemental Figures S1-S5. Supplemental Figure S1 - Correlation between rank and GC content calculated for the targets of every micro (mi)RNA family. Supplemental Figure S2 - Minimum hypergeometric (mHG) scores of human motifs versus mHG scores of Saccharomyces cerevisiae motifs. Supplemental Figure S3 - Predicted structures of selected mRNA targets. Supplemental Figure S4 - The enrichment of the Pumilio binding motif versus the enrichment of the miR-410 binding motif in validated Pumilio targets. Supplemental Figure S5 - Illustration of masking a region in a sequence. [file 1758-907X-1-17-S1.PDF]

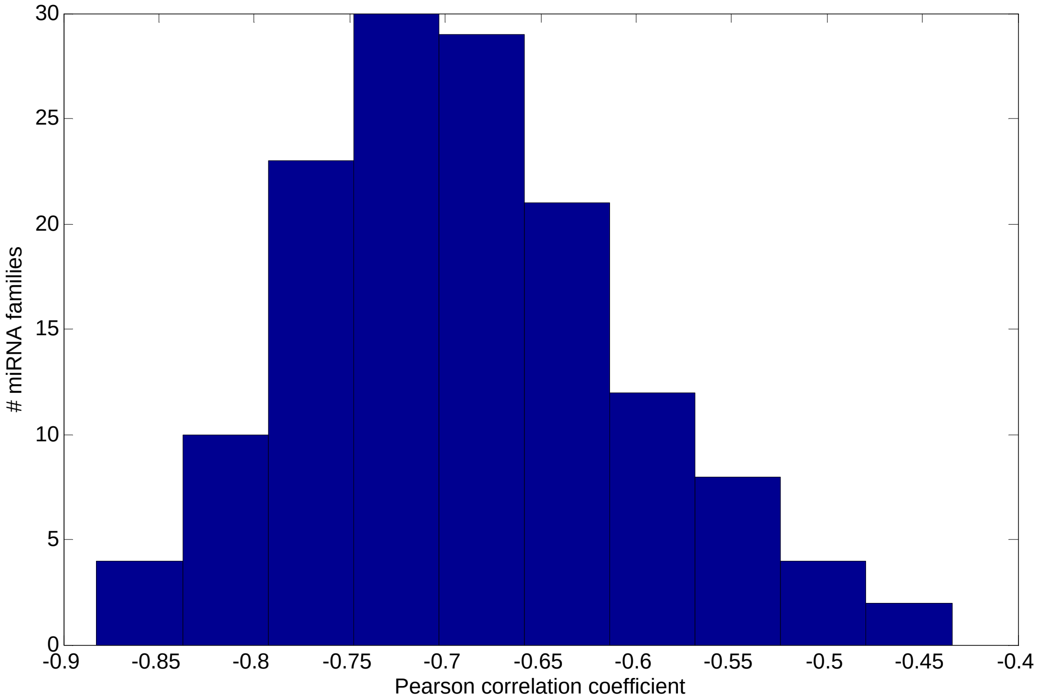

Supplemental Figure S1 - Correlation between rank and GC content calculated for the targets of every miRNA family

Given a miRNA family and its ranked list of targets, we calculated the correlation between the GC contents of these targets and their ranks. Pearson correlation coefficients were calculated for the targets of 153 conserved miRNA families, and are presented in the histogram above. The smallest correlation coefficient was found for miR-410 targets (-0.4336).

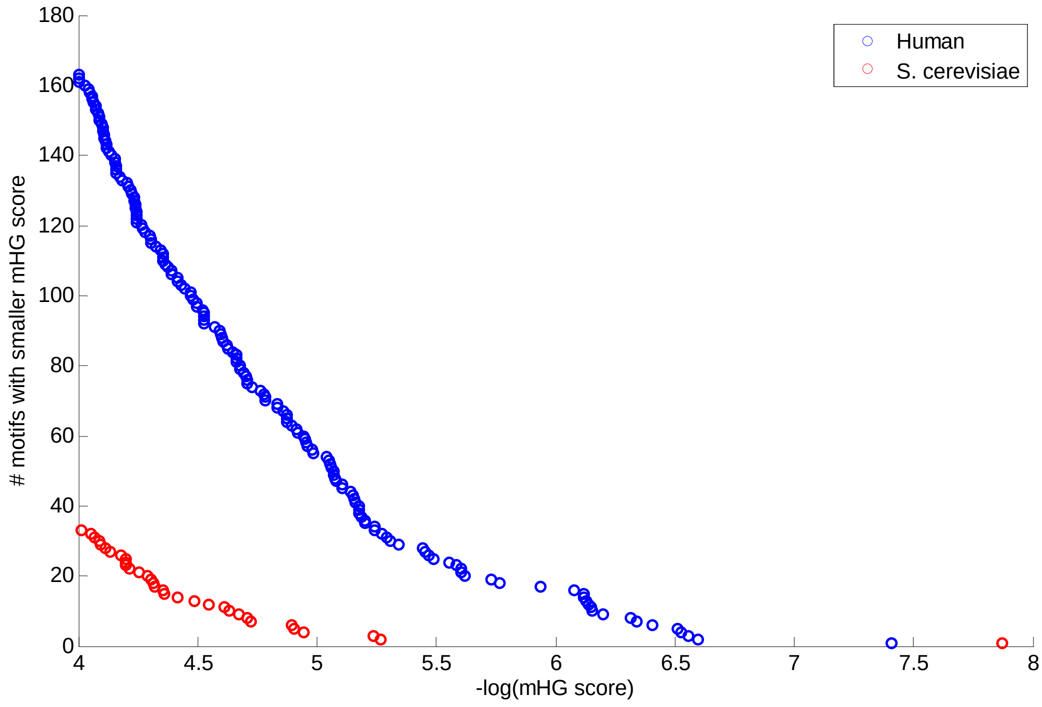

Supplemental Figure S2 - mHG scores of human motifs versus mHG scores of *S. cerevisiae* motifs

DRIM found 163 motifs enriched in human low accessible targets and 33 motifs enriched in *S. cerevisiae* low accessible targets. Their mHG scores are shown. DRIM returns motifs which have mHG score smaller than 10^{-4}.

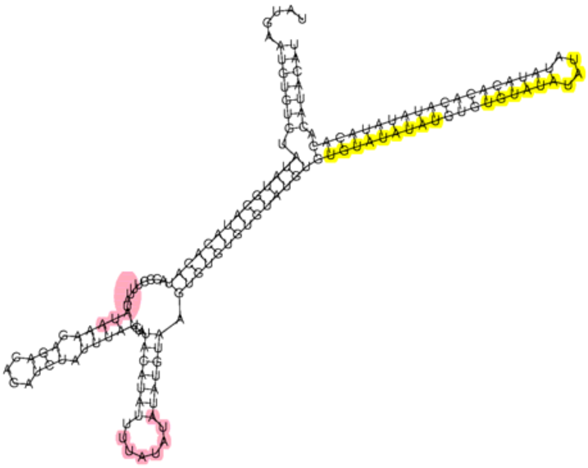

FAM120C

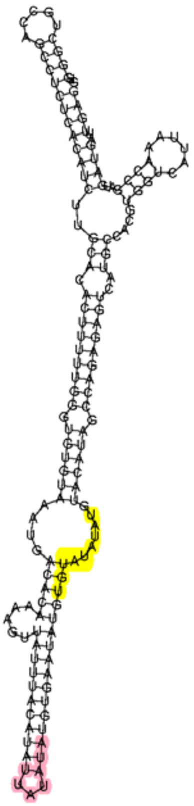

C14orf102

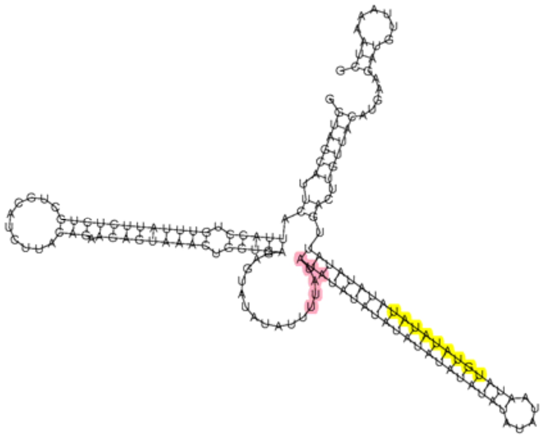

NRIP1

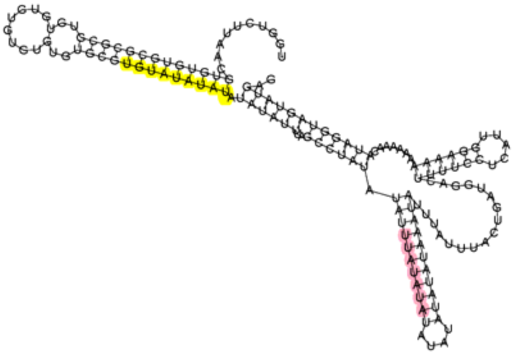

DGKG

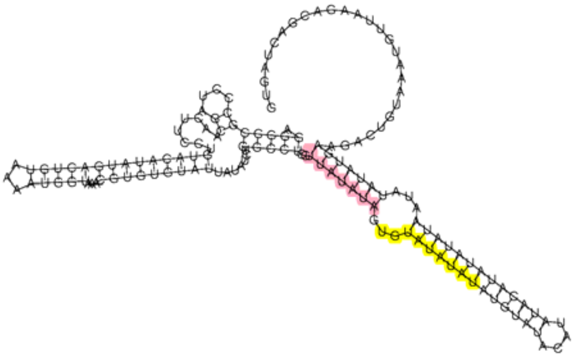

RAI1

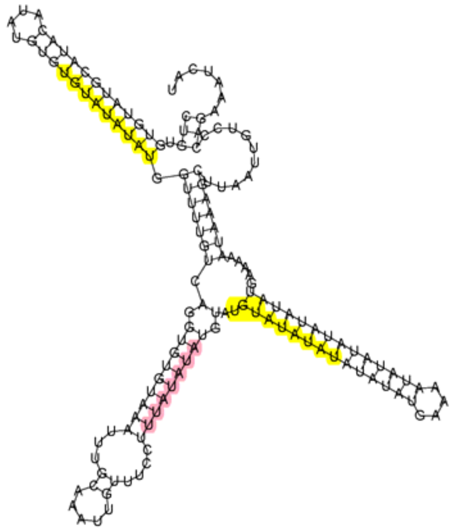

MCAM

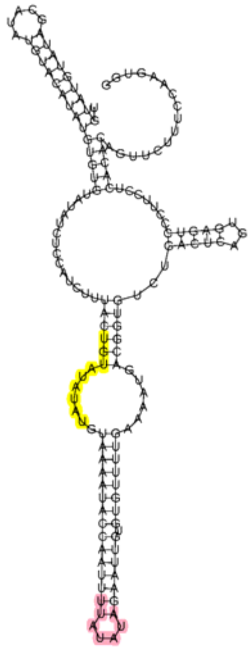

STARD13

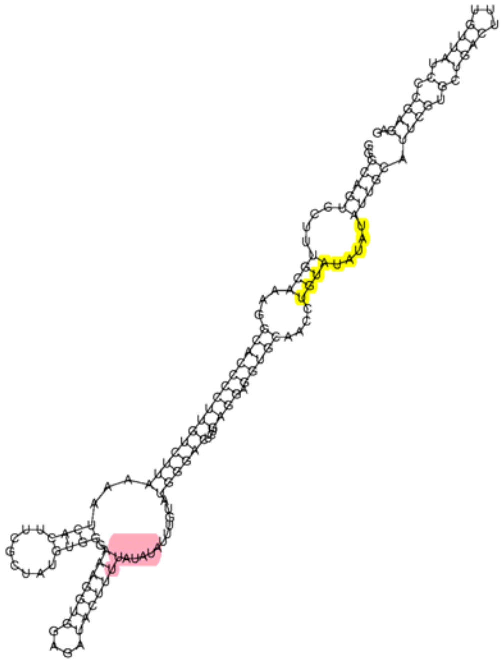

CELSR1

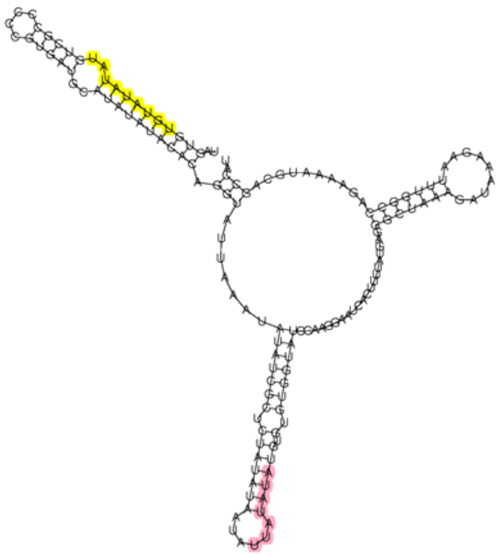

ZC3H7B

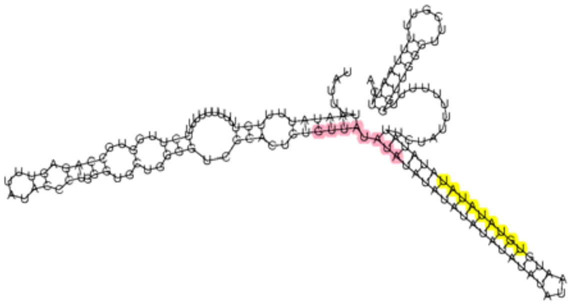

NDST1

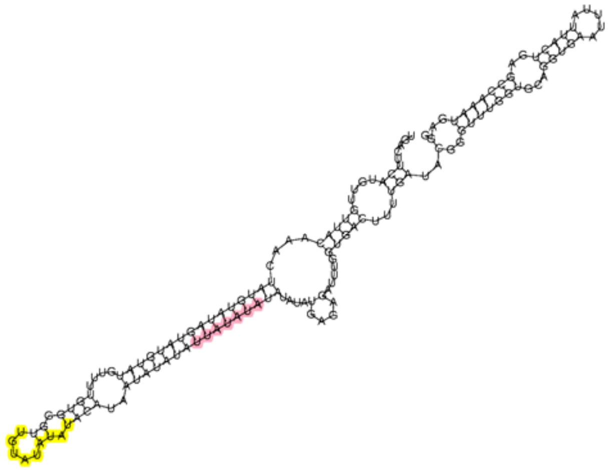

FZD5

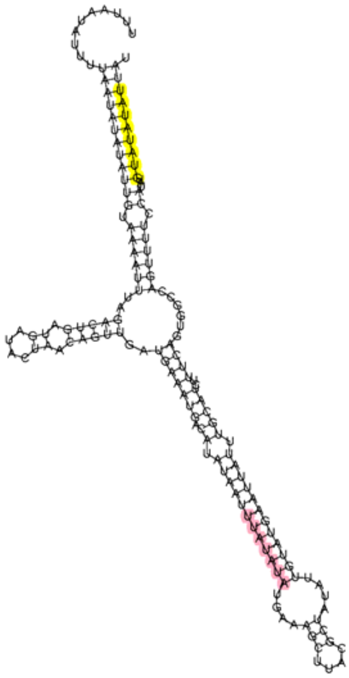

HACE1

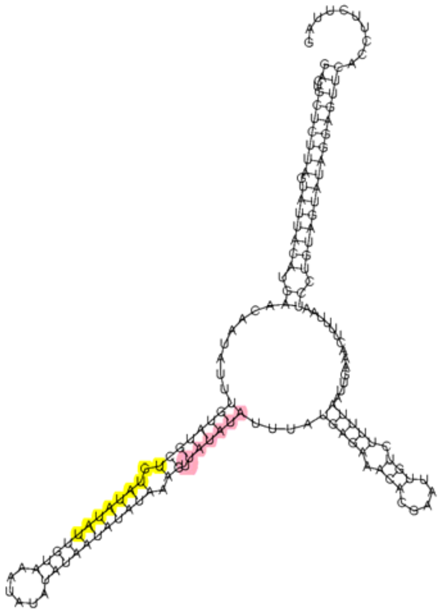

TEC

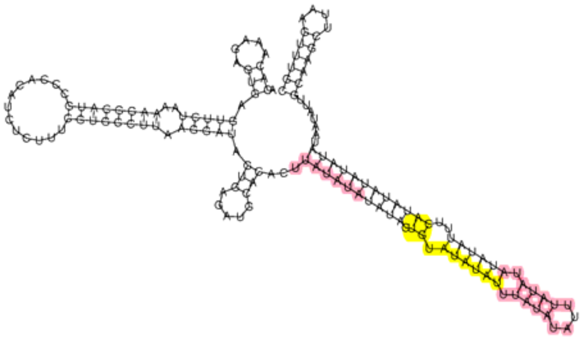

PRRX1

Supplemental Figure S3 - Predicted structures of selected mRNA targets

The structures of fourteen genes that are considered as low accessibility targets (according to the mHG statistics) and contain occurrences of the motif UGUAUAUAU (highlighted with yellow) and miR-410 binding sites (highlighted with pink) are shown. The targets are sorted according to their global accessibility scores (the upper left mRNA is the least accessible of all mRNAs shown). The gene symbol of each mRNA is indicated below its structure.

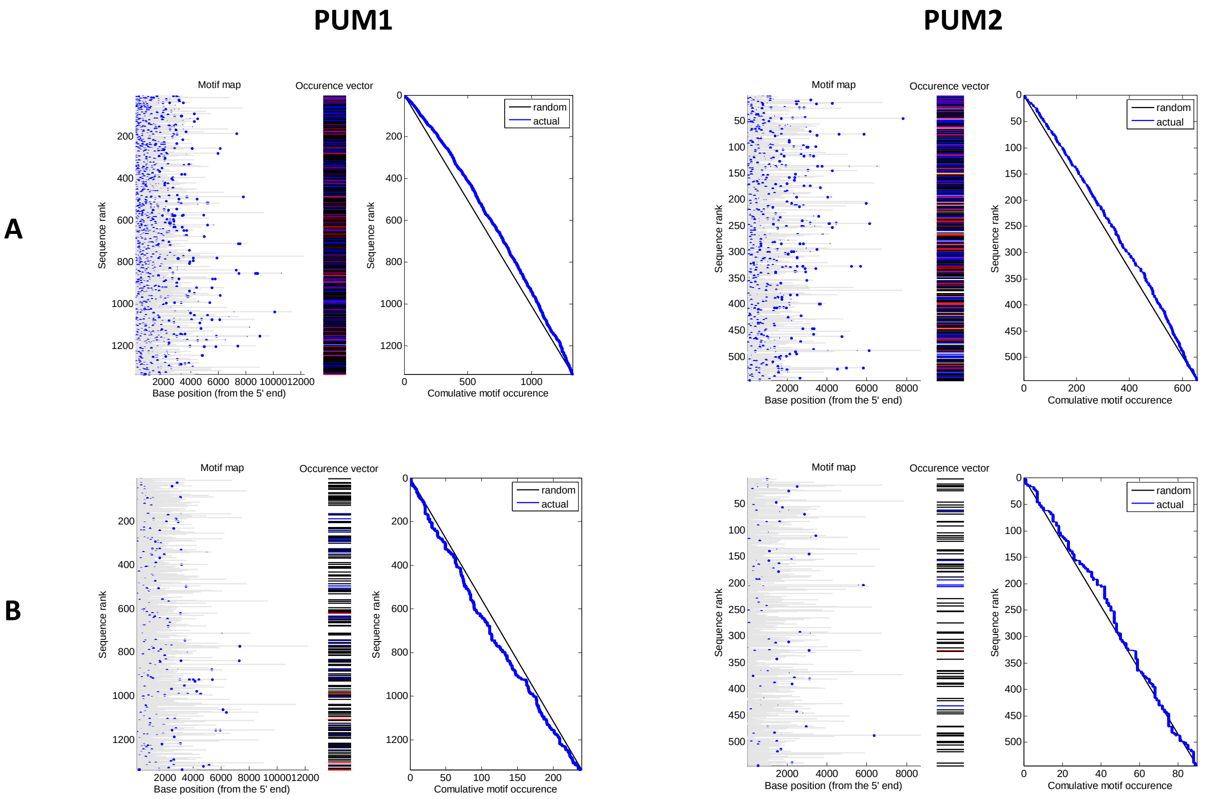

Supplemental Figure S4 - The enrichment of Pumilio binding motif versus the enrichment of miR-410 binding motif in Pumilio targets

PUM1 and PUM2 targets are ranked according to Pumilio binding affinity (in decreasing order). In each plot the occurrences of the motif among the sequences are shown on the left. In the middle, the occurrences vector plot illustrates the number of motif occurrences in each sequence (black for one occurrence, blue for two occurrences, red for more than two occurrences). The actual motif accumulated occurrences versus the expected motif accumulated occurrences in a random dataset are shown on the right.

(A) The enrichments of Pumilio binding motif (UGUAHAUA) among PUM1 targets (left) and PUM2 targets (right) are shown (p-values: $2.47 \cdot 10^{-9}$ and $2.7 \cdot 10^{-2}$, respectively).

(B) The enrichments of miR-410 binding motif (GUUAUAU) among PUM1 targets (left) and PUM2 targets (right) are shown (p-values: 0.86 and 0.48, respectively).

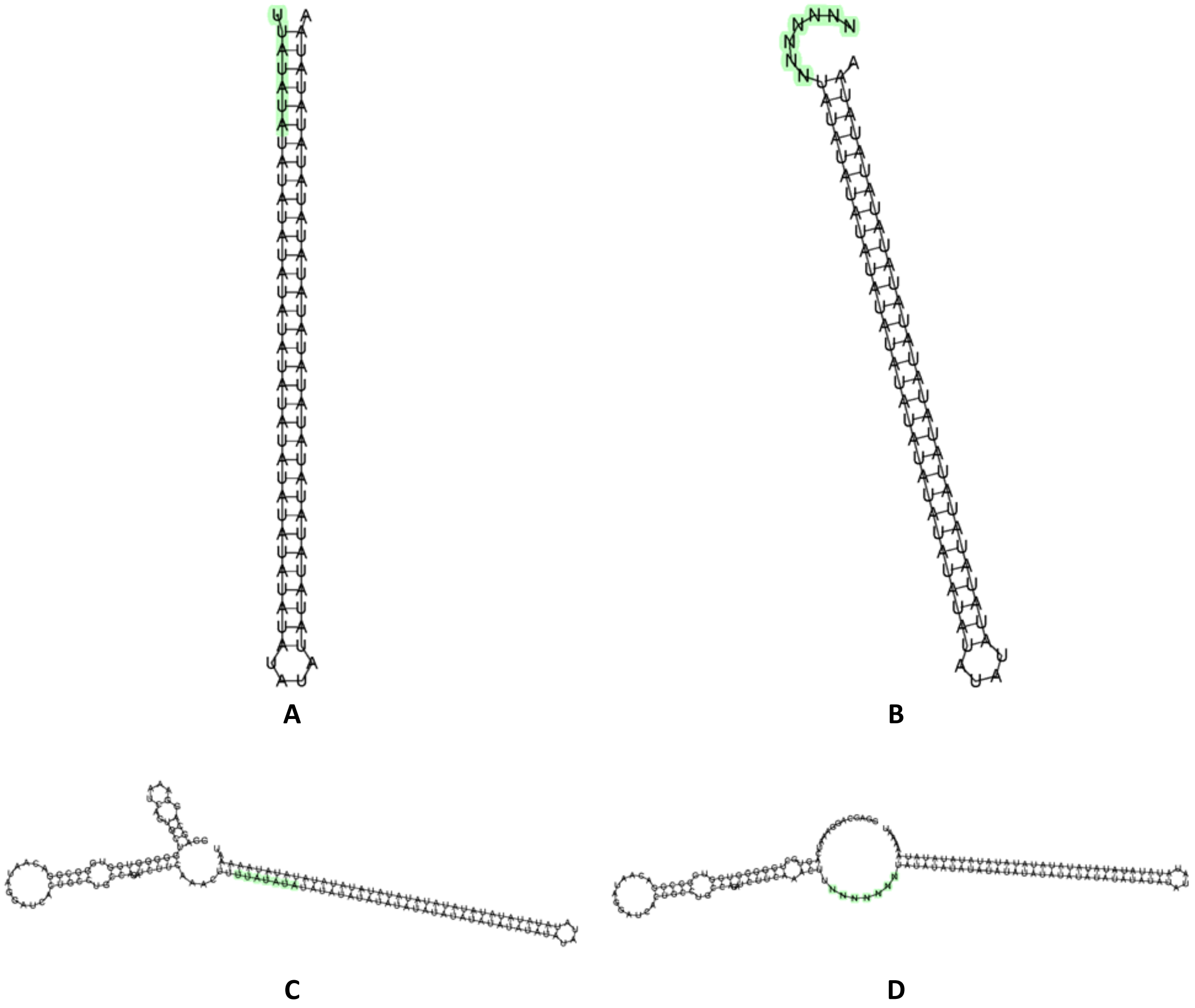

Supplemental Figure S5 - Illustration of masking a region in a sequence

(A), (C) The structures of the upper and lower sequences, respectively, as calculated by RNAfold.

(B), (D) The structures obtained after masking seven nucleotides in the upper and lower sequences, respectively (by replacing these nucleotides with N; the masking region is marked with light green), as calculated by RNA fold.
